# Opposing functions of β-arrestin 1 and 2 in Parkinson’s disease via microglia inflammation and Nprl3

**DOI:** 10.1038/s41418-020-00704-9

**Published:** 2021-03-08

**Authors:** Yinquan Fang, Qingling Jiang, Shanshan Li, Hong Zhu, Rong Xu, Nanshan Song, Xiao Ding, Jiaqi Liu, Miaomiao Chen, Mengmeng Song, Jianhua Ding, Ming Lu, Guangyu Wu, Gang Hu

**Affiliations:** 1 Jiangsu Key Laboratory of Neurodegeneration, Department of Pharmacology, Nanjing Medical University, 818 Tianyuan East Road, Nanjing, 211166 Jiangsu China; 2 Department of Pharmacology, Nanjing University of Chinese Medicine, 138 Xianlin Avenue, Nanjing, 210023 Jiangsu China; 3 Department of Pharmacology and Toxicology, Medical College of Georgia, Augusta University, 1459 Laney Walker Blvd., Augusta, GA 30912 USA

**Keywords:** Neuroscience, Neurological disorders

## Abstract

Although β-arrestins (ARRBs) regulate diverse physiological and pathophysiological processes, their functions and regulation in Parkinson’s disease (PD) remain poorly defined. In this study, we show that the expression of β-arrestin 1 (ARRB1) and β-arrestin 2 (ARRB2) is reciprocally regulated in PD mouse models, particularly in microglia. ARRB1 ablation ameliorates, whereas ARRB2 knockout aggravates, the pathological features of PD, including dopaminergic neuron loss, neuroinflammation and microglia activation in vivo, and microglia-mediated neuron damage in vitro. We also demonstrate that ARRB1 and ARRB2 produce adverse effects on inflammation and activation of the inflammatory STAT1 and NF-κB pathways in primary cultures of microglia and macrophages and that two ARRBs competitively interact with the activated form of p65, a component of the NF-κB pathway. We further find that ARRB1 and ARRB2 differentially regulate the expression of nitrogen permease regulator-like 3 (Nprl3), a functionally poorly characterized protein, as revealed by RNA sequencing, and that in the gain- and loss-of-function studies, Nprl3 mediates the functions of both ARRBs in microglia inflammatory responses. Collectively, these data demonstrate that two closely related ARRBs exert opposite functions in microglia-mediated inflammation and the pathogenesis of PD which are mediated at least in part through Nprl3 and provide novel insights into the understanding of the functional divergence of ARRBs in PD.

## Introduction

Parkinson’s disease (PD) is the second most common neurodegenerative disorder after Alzheimer’s disease (AD), and affects ~2–3% of the world’s population over the age of 65 [1, 2]. Although the etiology and pathogenic mechanism of PD are not fully understood, a variety of genetic factors and environmental exposures have been identified to contribute to the pathological progression of PD, and the possible mechanisms include the changes in dopamine metabolism, mitochondrial dysfunction, endoplasmic reticulum stress, impaired autophagy, and deregulated immunity [3]. Chronic neuroinflammation in the substantia nigra pars compacta (SNc), the progressive loss of dopaminergic (DA) neurons in SNc, and the presence of Lewy bodies in different nuclei of the nervous system are the neuropathological hallmarks of PD [4].

Microglia are the main immune cells of the brain and their activation-mediated inflammatory processes in PD have been investigated extensively [5, 6]. It is now increasingly apparent that sustained microglia activation is a major contributor to neuroinflammation and responsible for exacerbated neurodegeneration in PD [7, 8]. One of the characteristics of activated microglia is the enhanced production of pro-inflammatory cytokines, such as tumor necrosis factor α (TNFα), interleukin (IL) 1β, IL-6, and interferon-γ (IFN-γ), which are toxic to DA neurons [9–12]. Activated microglia, as well as concentration of pro-inflammatory factors, are increased in the SNc of PD patients [13–15]. Therefore, identification of the regulators involved in microglia activation may create new avenues for drug design in the treatment of PD.

β-Arrestins (ARRBs) are originally identified to mediate the desensitization and intracellular trafficking of G protein-coupled receptors (GPCRs) [16–21]. There are two ARRB isoforms, ARRB1 and ARRB2; they share 78% amino acid identity. It is now widely appreciated that both ARRBs may serve as important signal transducers and scaffolds to control multiple intracellular signaling cascades in a GPCR-dependent or independent fashion [22–25], with the inflammatory nuclear factor-κB (NF-κB) pathway being a well-studied example in which ARRBs may directly interact with pathway components [26–28].

There is considerable evidence supporting the diverse roles of ARRBs in the pathogenesis of central nervous system diseases, such as ischemia, AD, and multiple sclerosis [29–33]. Several recent studies have also implied a role for ARRB2 in PD. For example, ARRB2 in microglia mediates the functions of dynorphin/κ-opioid receptors in controlling endotoxin-elicited pro-inflammatory responses and protecting tyrosine hydroxylase (TH) positive neurons from inflammation-induced toxicity [34]. ARRB2 is involved in the development of L-DOPA-induced dyskinesia [35]. Studies from our laboratory have shown that ARRB2 negatively regulates the assembly and activation of NOD-like receptor protein-3 (NLRP3) via direct interaction in astrocytes, which contributes to the anti-neuroinflammatory effect of dopamine D2 receptors in PD [36]. However, noting is known about the functions of ARRB1 in the pathological progression of PD.

The purposes of this study are to investigate the possible functions of both ARRB1 and ARRB2 in the pathogenesis of PD and to elucidate the underlying mechanisms. We demonstrate that by virtue of their abilities to differentially regulate nitrogen permease regulator-like 3 (Nprl3) and the NF-κB and signal transducers and activators of transcription 1 (STAT1) pathways in microglia, ARRB1, and ARRB2 opposingly affect DA neuron degeneration and microglia inflammation both in vitro and in vivo. These data not only reveal novel functions and regulatory mechanisms of ARRBs in PD but also suggest a potential therapeutic approach for the disease.

## Results

### Differential expression of ARRBs in PD mouse models

**Fig. 1:**
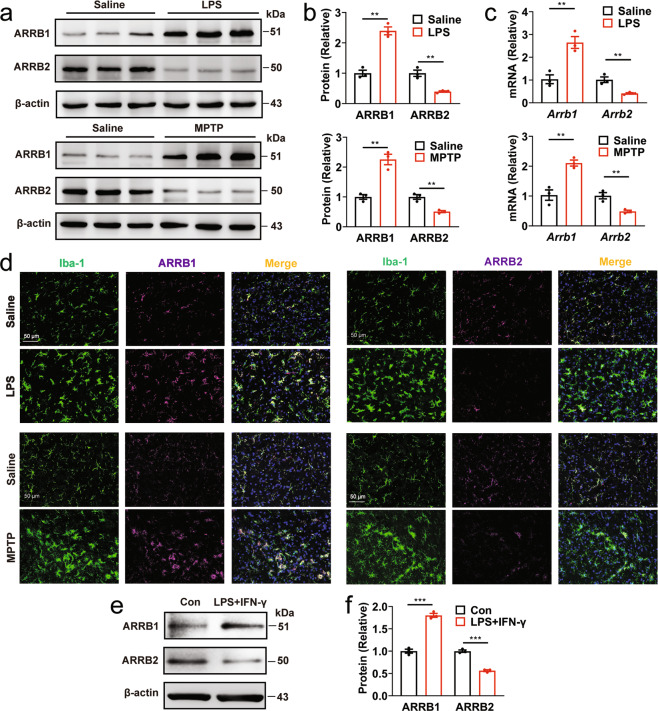
Expression of ARRB1 and ARRB2 in PD mouse models. **a** ARRB1 and ARRB2 expression in the SNc of LPS- and MPTP-induced PD mouse models. **b** Quantitative data shown in **a**. **c** mRNA of ARRB1 and ARRB2 in the SNc of PD mice measured by RT-PCR. **d** Expression of ARRB1 and ARRB2 in microglia (Iba-1) in the SNc of PD mouse models. Similar results were obtained in three separate experiments. **e** Expression of ARRB1 and ARRB2 in primary microglia after LPS plus IFN-γ stimulation. **f** Quantitative data shown in **e**. Quantitative data are mean ± s.e. (*n* = 3). **P < 0.01 and ***P < 0.001. Scale bars, 50 µm.

As an initial approach to define the functions of ARRBs in PD, we measured their expression in the midbrain of lipopolysaccharides (LPS)- and 1-methyl-4-phenyl-1,2,3,6-tetrahydropyridine (MPTP)-induced PD mouse models [37]. ARRB1 and its mRNA were increased by ~150%, whereas ARRB2 and its mRNA were decreased by more than 50%, in LPS-induced PD model (Fig. 1a–c). Such opposite regulation of ARRB1 and ARRB2 expression was also observed in MPTP-induced PD model (Fig. 1a–c). More interestingly, immunostaining showed that both ARRBs were highly expressed in ionized calcium binding adapter molecule 1 (Iba1)^+^ microglia (Fig. 1d), but barely in Glial fibrillary acidic protein (GFAP)^+^ astrocytes (Supplementary Fig. S1a) and neuronal nuclei (NeuN)^+^ neurons (Supplementary Fig. S1b) in the SNc of LPS- and MPTP**-**induced PD mice. Consistent with their expression in PD models in vivo, ARRB1 expression was markedly augmented, whereas ARRB2 expression was attenuated in primary cultures of microglia after LPS plus IFN-γ stimulation (Fig. 1e, f). These data suggest that the expression of ARRB1 and ARRB2, particularly in microglia, is differentially regulated in both inflammation- and toxin-induced mouse models of PD.

### Effects of ARRB knockout on DA neuron loss, microglia activation and neuroinflammation in PD models in vivo

**Fig. 2:**
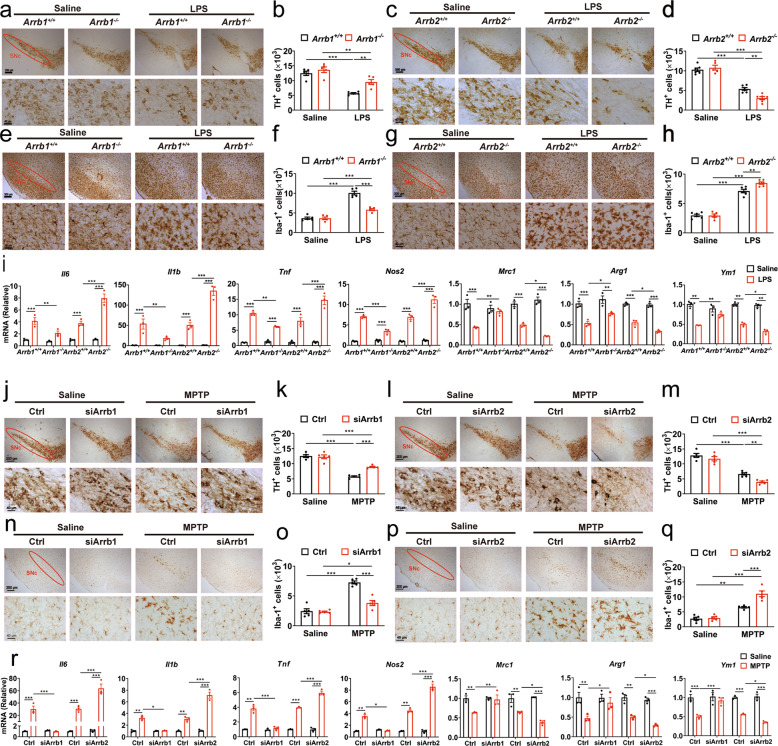
Effects of ARRB1 or ARRB2 depletion on neuron death and neuroinflammation in PD models. **a**–**h** Immunohistochemistry (**a**, **c**, **e** and **g**) and stereological counts (**b**, **d**, **f** and **h**) of TH^+^ DA neuron (**a**–**d**) and Iba-1^+^ microglia (**e**–**h**) in the SNc of LPS-induced PD models (*n* = 5). Scale bars, 200 µm (upper panels) or 40 µm (lower panels) in **a**, **c**, **e** and **g**. **i** mRNA levels of pro- and anti-inflammatory markers in the midbrain of PD mice (*n* = 3). **j**–**q** Immunohistochemistry (**j**, **l**, **n**, and **p**) and stereological counts (**k**, **m**, **o**, and **q**) of TH^+^ DA neuron (**j**–**m**) and Iba-1^+^ microglia (**n**–**q**) in the SNc of MPTP-induced PD models after AAV injection (*n* = 5). Scale bars, 200 µm (upper panels) or 40 µm (lower panels) in **j**, **l**, **n** and **p**. **r** mRNA levels of pro- and anti-inflammatory markers in the midbrain of PD mice after AAV injection (*n* = 3). Quantitative data are mean ± s.e. *P < 0.05, **P < 0.01, and ***P < 0.001.

We then used *Arrb1*^*−/−*^ and *Arrb2*^*−/−*^ knockout mice to generate PD mouse models by LPS or MPTP challenge and studied DA neuron loss and microglia activation in the SNc. Ablation of ARRB1 or ARRB2 was confirmed by immunoblotting and one isoform knockout did not affect the expression of the other isoform (Supplementary Fig. S2). As expected, LPS challenge caused remarkable DA neuron death and microglia activation as measured by staining with antibodies against TH and Iba-1, respectively, in wild-type (WT), *Arrb1*^*−/−*^ and *Arrb2*^*−/−*^ mice. However, LPS-induced neuron loss and microglia activation were significantly alleviated in *Arrb1*^*−/−*^− mice, but exacerbated in *Arrb2*^*−/−*^ mice, as compared with those in WT mice (Fig. 2a–h).

We next determined the effects of ARRB knockout on neuroinflammation by measuring the expression of inflammatory markers in the mouse midbrain. All pro-inflammatory markers tested, including *Il6, Il1b, Tnf*, and *Nos2* genes and inducible nitric-oxide synthase (iNOS, encoded by *Nos2*), were significantly decreased, whereas anti-inflammatory markers, including *Arg1, Ym-1* and *Mrc1* genes and CD206 (encoded by *Mrc1*), were increased in *Arrb1*^*−/−*^ mice after LPS challenge, as compared with those in WT mice. In marked contrast, the pro-inflammatory markers were enhanced and the anti-inflammatory markers were reduced in *Arrb2*^*−/−*^ mice as compared with those in WT mice (Fig. 2i and Supplementary Fig. S3a–d). Similar to the results observed in LPS-induced PD models, knockout of ARRB1 and ARRB2 produced opposite effects on DA neuron loss, microglia activation, and neuroinflammation in MPTP-induced PD mouse models in vivo (Supplementary Fig. S3e–q).

To define the role of microglial ARRBs in the PD mouse model, AAVs carrying the microglia-specific promoter F4/80 (Supplementary Fig. S4a) were used to deliver siRNA to knock down ARRB1 or ARRB2 in microglia as confirmed by immunostaining (Supplementary Fig. S4b). Similar to the results observed in *Arrb1*^*−/−*^ and *Arrb2*^*−/−*^ mice, MPTP-induced loss of DA neurons, activation of microglia and neuroinflammation all were significantly alleviated by AAV-mediated knockdown of microglial ARRB1, but exacerbated by knockdown of microglial ARRB2 in vivo (Fig. 2j–r and Supplementary Fig. S3r–u). These results indicate that microglial ARRB1 and ARRB2 play opposite roles in PD mouse models.

### Effects of ARRB depletion on microglia-induced DA neuron damage

**Fig. 3:**
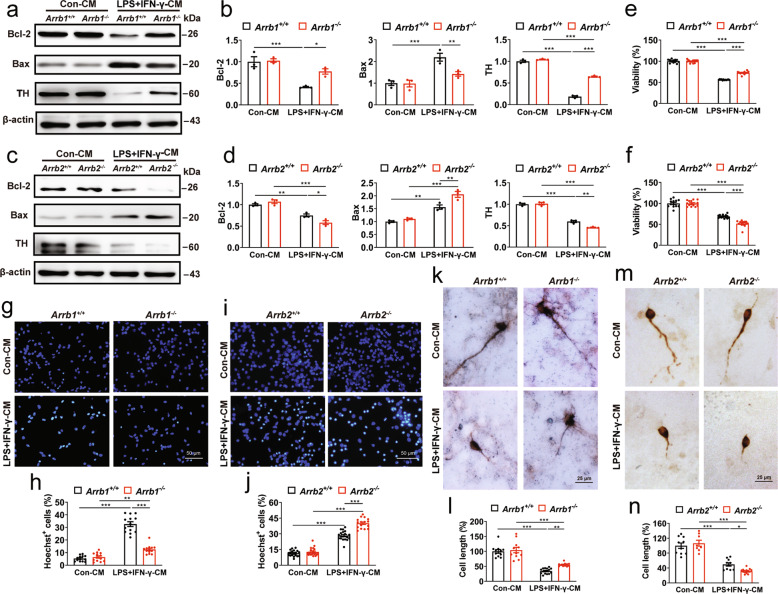
Effects of ARRB1 and ARRB2 knockout on microglia-induced DA neuron damage. **a**–**d** Expression of Bcl-2, Bax and TH in DA neurons treated with CMs of WT, ARRB1 knockout or ARRB2-deficient microglia. **e** and **f** The viability of DA neurons. **g**–**j** Nuclear morphology (**g**–**i**) and Hoechst-positive DA neurons (**h**–**j**). Scale bars, 50 µm. **k**–**n** Morphology of DA neurons (**k**–**m**) and TH^+^ cell neurite length (**l**–**n**). Scale bars, 25 µm. Quantitative data are mean ± s.e. (*n* = 3). *P < 0.05, **P < 0.01, and ***P < 0.001.

To define if the effects of ARRBs on DA neuron loss were indeed caused by their actions on microglia activation as observed in the PD mouse models in vivo, we measured the effects of conditioned medium (CM) collected from microglia with or without LPS + IFN-γ treatment on DA neuron apoptosis, death and survival in vitro. The CM from microglia treated with LPS + IFN-γ strongly lowered the expression of anti-apoptotic Bcl-2, but elevated the expression of pro-apoptotic Bax in the neurons (Fig. 3a–d) and reduced the viability of the neurons (Fig. 3e, f). The neurons exhibited apoptotic features, including chromatin condensation and nuclear fragmentation (Fig. 3g–j). The CM from microglia treated with LPS + IFN-γ also decreased the expression of TH (Fig. 3a–d) and shrunk the length of DA neuron axons (Fig. 3k–n). All of these deleterious effects on the DA neurons were clearly mitigated by the CM from ARRB1 knockout microglia, but intensified by the CM from ARRB2 knockout microglia (Fig. 3). These results indicate that ARRB1 knockout can rescue, whereas ARRB2 depletion further amplify, the DA neuron damage induced by microglia inflammatory responses.

### Functions of ARRBs in inflammatory response of primary microglia and macrophages

**Fig. 4:**
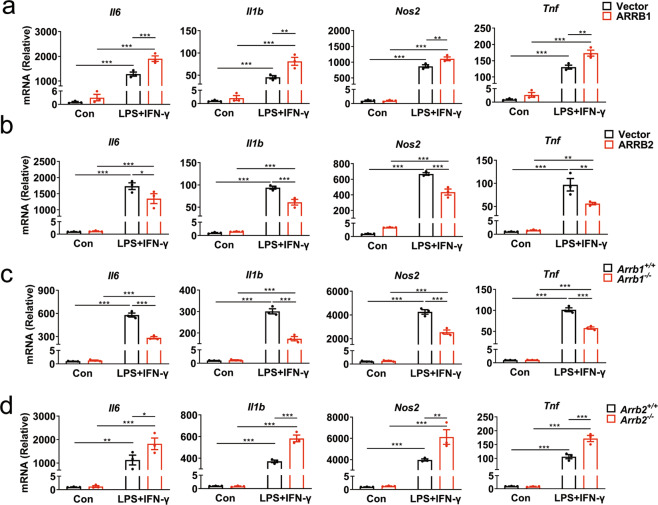
Effects of overexpression and depletion of ARRB1 or ARRB2 on microglia-mediated inflammation. Levels of pro-inflammatory gene transcripts in microglia transfected with ARRB1 (**a**) or ARRB2 (**b**) after LPS plus IFN-γ stimulation for 6 h. Pro-inflammatory gene expression in ARRB1 (**c**) and ARRB2 (**d**) knockout microglia. Quantitative data are mean ± s.e. (*n* = 3). **P < 0.01 and ***P < 0.001.

The gain- and loss-of-function approaches were used to further study the roles of ARRBs in microglia-mediated inflammation in response to LPS plus IFN-γ stimulation. In the gain-of-function studies, ARRB1 overexpression (Supplementary Fig. S5a, b) significantly promoted, whereas ARRB2 overexpression (Supplementary Fig. S5c, d) reduced, the expression of pro-inflammatory marker genes (*TNF-α*, *IL-6*, *IL-1β,* and *iNOS*) in microglia (Fig. 4a, b). In the loss-of-function studies, ARRB1 knockout significantly inhibited (Fig. 4c), whereas ARRB2 knockout raised, the expression of the pro-inflammatory markers (Fig. 4d).

**Fig. 5:**
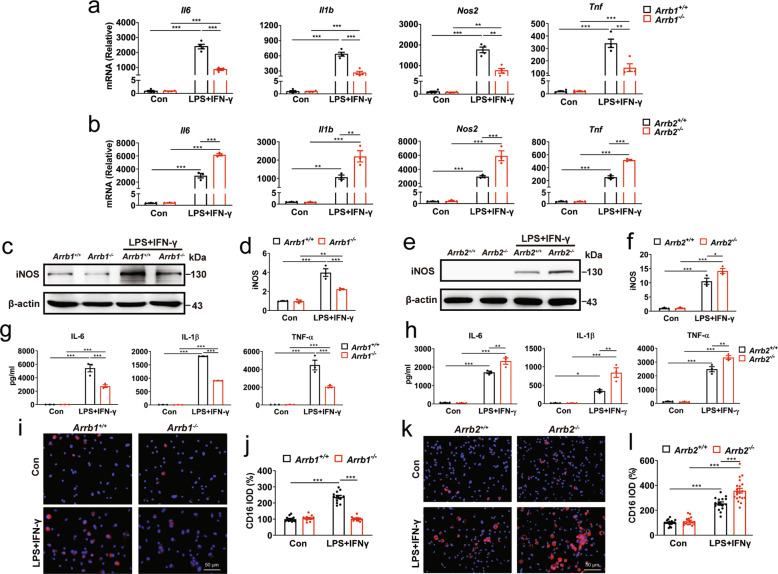
Effects of ARRB1 and ARRB2 depletion on inflammation in macrophages. **a** and **b** Levels of pro-inflammatory gene in ARRB1 and ARRB2 knockout BMDMs with or without LPS plus IFN-γ stimulation for 6 h. **c**–**f** iNOS expression in ARRB1 and ARRB2 knockout BMDMs after stimulation for 24 h. **g** and **h** IL-6, IL-1β, and TNF-α expression in supernatants of ARRB1 and ARRB2 knockout BMDMs after stimulation for 24 h. **i**–**l** CD16 expression detected by immunofluorescence. Scale bars, 50 μm. Quantitative data are mean ± s.e. (*n* = 3). *P < 0.05, **P < 0.01, and ***P < 0.001.

As microglia and macrophages have similar properties in mediating inflammation [38–40], bone marrow-derived macrophages (BMDMs) were used to confirm the functions of ARRBs in microglia-mediated inflammation. Similar to the results observed in microglia, ablation of ARRB1 markedly lowered the expression of pro-inflammatory marker genes and iNOS in BMDMs after LPS plus IFN-γ stimulation (Fig. 5a, c, d), whereas knockout of ARRB2 enhanced the expression of pro-inflammatory markers (Fig. 5b, e, f). Furthermore, ARRB1 knockout decreased, whereas ARRB2 knockout increased, the release of pro-inflammatory cytokines (IL-6, IL-1β, and TNF-α) as measured by ELISA (Fig. 5g, h). Immunofluorescent imaging showed that ARRB1 depletion attenuated the expression of CD16, a pro-inflammatory marker, in BMDMs upon stimulation with LPS plus IFN-γ (Fig. 5i, j). In contrast, ARRB2 depletion enhanced CD16 expression (Fig. 5k, l) in BMDMs. These data demonstrate that ARRB1 and ARRB2 expression levels may directly control the inflammatory responses in a contrary manner in microglia and macrophages

### Roles of ARRBs in the activation of inflammatory pathways and their interaction with p65

**Fig. 6:**
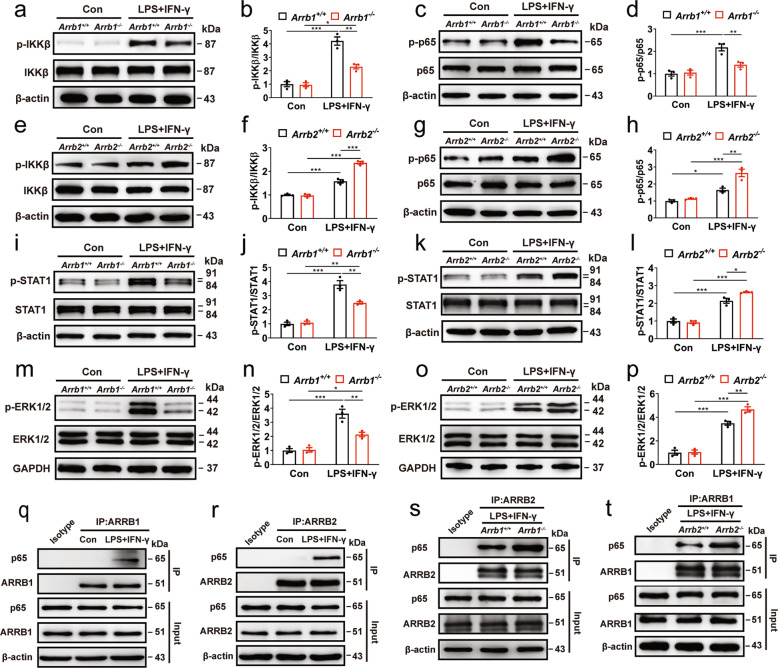
Effects of knockout of ARRB1 or ARRB2 on activation of NF-κB, STAT1 and ERK1/2 pathways. Activation of IKKβ (**a**, **b**, **e** and **f**), p65 (**c**, **d**, **g** and **h**), STAT1 (**i**–**l**) and ERK1/2 (**m**–**p**) in ARRB1 or ARRB2 knockout BMDMs with or without LPS plus IFN-γ stimulation for 2 h. **q**–**r** Interaction of ARRB1 or ARRB2 with p65. BMDMs were stimulated with LPS plus IFN-γ for 1 h and the cell lysates were immunoprecipitated with antibodies against ARRB1 (**q**) or ARRB2 (**r**). **s** Effect of ARRB1 knockout on ARRB2 interaction with p65. **t** Effect of ARRB2 knockout on ARRB1 interaction with p65. Blots shown in **m**–**p** are representatives of three independent experiments. Quantitative data are mean ± s.e. (*n* = 3). *P < 0.05, **P < 0.01, and ***P < 0.001.

As activation of the NF-κB and STAT1 pathways plays an essential role in inflammatory responses [41–43] and ARRBs regulate extracellular signal-regulated kinase (ERK) 1/2 activation by GPCRs [44, 45], we determined if ARRBs could regulate these all three pathways. ARRB1 knockout significantly inhibited, whereas ARRB2 knockout stimulated, the activation of inhibitor of NF-κB (IκB) kinase β (IKKβ) and p65 in the NF-κB pathway in BMDMs treated with LPS plus IFN-γ (Fig. 6a–h). STAT1 and ERK1/2 activation were also impaired by ARRB1 knockout (Fig. 6i, j, m, n), but strengthened by ARRB2 knockout (Fig. 6k, l, o, p) in BMDMs. These results suggest that ARRB1 and ARRB2 may differentially regulate the activation of the NF-κB, STAT1, and MAPK pathways.

ARRBs have been shown to interact with three molecules, IκBα, IKKα, and ΙΚΚβ [46–48] in the NF-κB pathway. To determine if ARRBs could bind other molecules in this pathway, we measured their interaction with p65. ARRB1 and ARRB2 were found to robustly interact with p65 in co-immunoprecipitation (co-IP) assays. More interestingly, both interactions fully depended on inflammatory stimulation (Fig. 6q, r). Furthermore, knockout of one ARRB isoform clearly potentiated the interaction of the other isoform with p65 (Fig. 6s, t). These results suggest that two ARRBs physically associate with p65 likely in a competitive fashion.

### Nprl3 as a novel effector of ARRBs in microglia

To elucidate the molecular mechanisms underlying the function of ARRBs, we measured the effects of their phosphorylation and ubiquitination, which are known to control their functions in GPCR trafficking and signaling [49–52], on microglial neuroinflammation, by measuring the production of inflammatory factors. Expression of ARRB1 phosphorylation mutants (S412A and S412D), ARRB2 phosphorylation mutants (S361A/T383A and S361D/T383D), ARRB1-Ub, and ARRB2-Ub produced similar effects on the expression of pro-inflammatory marker genes, as compared with their wild type counterparts (Supplementary Fig. S6). These data suggest that phosphorylation and ubiquitination unlikely play a major role in ARRB-mediated inflammatory responses.

**Fig. 7:**
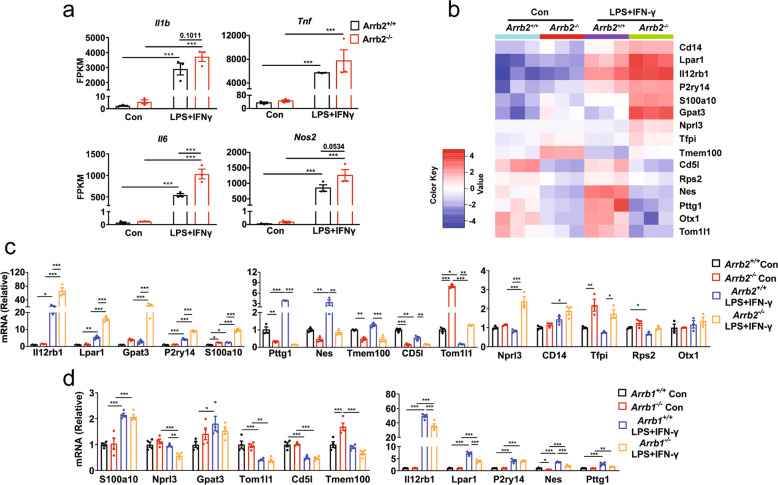
Effects of ARRB1 and ARRB2 on Nprl3 expression in microglia. **a** Normalized expression of pro-inflammatory markers measured by RNA-seq in microglia from WT and *Arrb2*^*−/−*^ mice with or without LPS plus IFN-γ treatment for 6 h. **b** Heatmap of inflammatory genes in microglia from WT and *Arrb2*^*−/−*^ mice. The genes shown are differentially expressed (padj ≤ 0.05, log2-fold change ≥1 or ≤ −1) in WT and *Arrb2*^*−/−*^ mice. **c** Expression of 15 genes shown in **b** were measured by RT-PCR. **d** Gene expression in ARRB1 knockout microglia. Quantitative data are mean ± s.e. (*n* = 3). *P < 0.05, **P < 0.01, and ***P < 0.001.

To identify the effectors acting downstream of ARRBs, RNA sequencing (RNA-seq) was performed to compare genome-wide transcriptional profiles in microglia from WT or *Arrb2*^*−/−*^ mice in response to inflammatory stimulation (Supplementary Fig. S7a). This strategy identified 130 genes upregulated and 56 genes downregulated in *Arrb2*^*−/−*^ mice, as compared with WT mice (Supplementary Fig. S7b). Analysis of the enriched biological processes showed that upregulated genes were related to positive regulation of immune responses, whereas downregulated genes associated with negative regulation (Supplementary Fig. S7b). Analysis of the enriched KEGG pathways also showed that upregulated genes were linked to inflammatory and immunological responses (Supplementary Fig. S7c). Consistent with our results above (Fig. 4d), the RNA-seq data showed that the expression of pro-inflammatory genes, including *Il1b, Tnf*, *Il6,* and *Nos2*, was increased in *Arrb2*^*−/−*^ mice as compared with WT mice (Fig. 7a).

Based on the RNA-seq data, 15 inflammation-related genes were clearly changed in *Arrb2*^*−/−*^ mice as compared with WT mice (Fig. 7b), and 11 of them (*Il12rb1, Lpar1, Gpat3, P2ry14, S100a1, Pttg1, Nes, Tmem100, CD5l, Tom1l1*, and *Nprl3*) were confirmed by RT-PCR (Fig. 7c). To determine if ARRB1 could alter the expression of these 11 gens, we measured the effects of ARRB1 knockout in microglia. Among these 11 genes, 3 genes, including *Il12rb1, Lpar1* and *Nprl3* which were increased in microglia from *Arrb2*^*−/−*^ mice, were decreased in ARRB1-depleted microglia (Fig. 7d) as measured by RT-PCR.

**Fig. 8:**
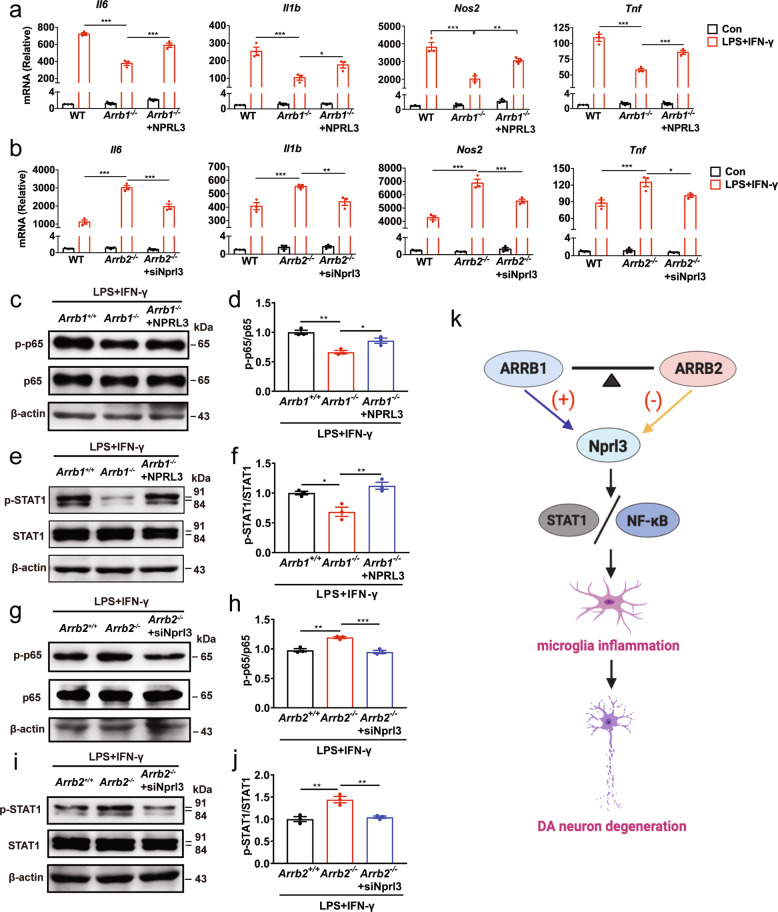
Effects of overexpression and knockdown of Nprl3 on ARRB-mediated microglia inflammation. Inflammatory gene expression in microglia from WT and ARRB knockout mice after transfection with either NPRL3 for 24 (**a**) or Nprl3 siRNA for 48 h (**b**). Activation of p65 (**c**, **d**, **g** and **h**) and STAT1 (**e**, **f**, **i** and **j**) in microglia after Nprl3 overexpression (**c**–**f**) or knockdown (**g**–**j**) as above. **k** A schematic diagram showing the opposite roles of ARRB1 and ARRB2 in microglia-mediated inflammation and DA neuron degeneration via regulating Nprl3 and the inflammatory STAT1 and NF-κB pathways (see text for details). +, stimulatory; −, inhibitory. Quantitative data are mean ± s.e. (*n* = 3). *P < 0.05, **P < 0.01, and ***P < 0.001.

Il12rb1 and Lpar1 are receptors for IL-12 and lysophosphatidic acid (LPA), respectively, and both are well known to regulate inflammatory responses [53–56]. The functions of Nprl3, however, are poorly studied and thus, it was selected to be studied in microglia activation. As our data demonstrated that Nprl3 was downregulated in *Arrb1*^*−/−*^ mice and upregulated in *Arrb2*^*−/−*^ mice, we determined the effects of Nprl3 overexpression in ARRB1-depleted microglia and the effects of Nprl3 knockdown in ARRB2-depleted microglia on inflammatory responses. Transient expression of Nprl3 (Supplementary Fig. S8a, b) enhanced the expression of pro-inflammatory marker genes (*Il6, Il1b, Tnf*, and *Nos2*), as well as the activation of p65 and STAT1 in ARRB1-knockout microglia, as compared with cells transfected with control vectors (Fig. 8a, c–f). siRNA-mediated Nprl3 knockdown (Supplementary Fig. S8c, d) inhibited the expression of pro-inflammatory marker genes and the activation of p65 and STAT1 in ARRB2-knockout microglia (Fig. 8b, g–j). These results suggest that Nprl3 is a novel effector, acting downstream of both ARRBs and mediating their functions in microglia inflammatory responses and activation of the NF-κB and STAT1 pathways.

## Discussion

In this study, we have demonstrated that ARRB1 and ARRB2, two closely related ARRBs, display functional antagonism in the pathogenesis of PD (Fig. 8k) which is mediated through their distinct actions on microglia inflammatory responses. We first found that the expression of ARRB1 and ARRB2 was adversely regulated in the SNc and microglia of PD mouse models. We then used *Arrb1*^*−/−*^ and *Arrb2*^*−/−*^ mice and microglia-specific depletion of ARRBs to demonstrate that ARRB1 ablation significantly ameliorated, whereas knockout of ARRB2 exaggerated, DA neuron degeneration, microglia activation, and neuroinflammation in two PD mouse models in vivo. The opposing functions of ARRB1 and ARRB2 were also observed in microglia-mediated DA neuron damage, inflammation, and the activation of inflammatory signaling pathways in the gain- and loss-of-function studies using primary cell cultures in vitro. These data demonstrate that the expression of individual ARRBs as well as their expression ratio, specifically in microglia, is a crucial tipping point to control microglia inflammatory responses which in turn affect DA neuron degeneration and eventually the development of PD (Fig. 8k).

We have also identified that Nprl3 is a novel effector, acting downstream of ARRBs and mediating their opposite effects on microglia inflammation. Nprl3 is a component of the gap activity towards rags 1 complex and regulates mTOR complex 1 signaling; it is associated with the pathogenesis of epilepsy and cancer [57–59]. However, its physiological functions remain largely unknown. In the current study, Nprl3 was identified as an effector of ARRBs by RNA-seq analysis of genome-wide transcriptional profiles, which was further confirmed by its ability to control the functions of ARRBs in microglia-mediated inflammatory responses, including the expression of pro-inflammatory factors and activation of the NF-κB and STAT1 pathways. It is worth noting that ARRB1 and ARRB2 competitively interact with p65 and the interaction is completely dependent on inflammatory stimulation, suggestive of p65 activation-dependent interaction. Previous studies have revealed that the effects of ARRBs on the activation of the NF-κB pathway depend on stimuli and cell types studied [28, 60] and the underlying mechanisms may involve direct interaction with IκBα, IKKα, and ΙΚΚβ [46–48]. Our data not only suggest a novel mechanism by which ARRBs activate the NF-κB pathway, but also imply distinct functions of ARRBs in PD being attributable to their different abilities to interact with p65.

Our data presented in this study have demonstrated that, to the best of our knowledge, ARRB1 and ARRB2 are the only and first pair of closely related isoforms which produce completely opposing functions in both in vivo and in vitro models of PD. Based on these data, we can conclude that the normal function of ARRB1 in microglia is stimulatory, whereas the normal function of ARRB2 is inhibitory, with respective to the expression of Nprl3, the activation of the STAT1 and NFκB pathways, inflammatory responses, and pathologic progression of PD (Fig. 8k). As such, these data imply a potential therapeutic intervention, using genetic and/or pharmacological approaches to inhibit ARRB1 function and enhance ARRB2 function simultaneously,

It is interesting to note that distinct expression and function of ARRB1 and ARRB2 have been observed in other animal and cellular settings [60, 61]. For example, in an experimental autoimmune encephalomyelitis (EAE) mouse model, ARRB1 knockout alleviates disease phenotypes [32], whereas ARRB2 deficiency exacerbates EAE symptoms [33]. In a cellular model, ARRB1 downregulation increases, whereas ARRB2 depletion reduces, angiotensin II receptor-mediated activation of extracellular signal-regulated kinases 1 and 2 [62, 63], which are contradictory to our results in BMDMs in response to inflammatory stimulation. Similar to the results observed in this study, both ARRB1 and ARRB2 interact with NLRP3 inflammasome, but they produce functionally contrary effects on the inflammasome activation [64, 65]. These data, together with our current study, demonstrate the extreme complexity of ARRB-mediated functions under different physiological and pathological conditions.

In summary, this study reveals for the first time important, but opposite, roles played by ARRB1 and ARRB2 in the microglia-mediated inflammation and pathogenesis of PD. Our results provide novel insights into the understanding of the functional divergence of ARRBs in PD and may aid in the development of drugs for the treatment of PD.

## Methods and materials

### Antibodies and reagents

Antibodies against ARRB1 (cat# 12697), ARRB2 (3857), iNOS (13120), p-p65 (3033), p65 (8242), p-IKKβ (2697), IKKβ (8943), p-STAT1 (7649), STAT1 (14995), p-ERK1/2 (4370), ERK1/2 (9102), and Bax (2772) were purchased from Cell Signaling Technologies; CD206 (AF2534) antibodies and all ELIZA kits from R&D Systems; Bcl-2 (40639) antibodies from Signalway Antibody; TH (T1299) and β-actin (A1978) antibodies, LPS, MPTP, poly-L-lysine (PLL), bovine serum albumin (BSA), Triton X-100, and Hoechst 33324 from Sigma; ARRB1 (53780) and ARRB2 (514791) antibodies from Santa Cruz; Nprl3 (NBP1-88447) antibodies from Novus Biologicals; Iba1 (019-19741) antibodies from Wako, Japan; CD16 (553142) antibodies from BD Biosciences; NeuN (ab177487) and GFAP (ab7260) antibodies from Abcam; Alexa-conjugated secondary antibodies, Lipofectamine RNAi MAX, Lipofectamine 3000, TRIzol reagent and DAPI from Invitrogen; trypsin, EDTA, Dulbecco’s modified eagle medium (DMEM), DMEM/F-12 medium, fetal bovine serum (FBS), streptomycin, penicillin, sodium pyruvate, neurobasal medium, B27, and glutamine from Gibco; granulocyte-macrophage colony stimulating factor (GM-CSF) and IFN-γ from PeproTech; protease inhibitor cocktail and FastStart Universal SYBR Green Master from Roche; TaKaRa Master Mix from TaKaRa, Japan; DAB staining system from Boster, China; cell counting kit-8 (CCK-8) from Dojindo, China; protein-A/G beads from Thermo Scientific; the GFP-tagged ARRB1 and Nprl3 plasmids and all adeno-associated viruses (AVVs) from GeneChem Co.; all siRNAs from GenePharma, China.

### Animals and PD models

*Arrb1*^*−/−*^ mice were purchased from the Jackson Laboratory (Las Vegas, NV) and *Arrb2*^*−/−*^ mice kindly provided by Dr. Gang Pei (Tongji University, Shanghai, China). C57BL/6J WT mice were from the Animal Core Faculty of Nanjing Medical University. All mice were allowed access to food and water ad libitum and maintained at 22–24 °C with a 12 h light/dark cycle. All animal experiments were carried out in compliance with the ethical regulations and approved by Institutional Animal Care and Use Committee of the Nanjing Medical University Experimental Animal Department.

The LPS- and MPTP-induced PD models were developed as described previously [36, 66]. Briefly, mice (male, 3-4 months old) were randomly divided into groups (*n* = 11 for each genotype) and microinjected bilaterally with LPS (0.5 µg in 1 µl of saline, Sigma) at the SNc (AP: −3.0 mm; ML: ± 1.3 mm; DV: −4.2 mm) at a rate of 0.2 μl/min in a stereotaxic apparatus or administered with MPTP (20 mg/kg, i.p., Sigma) four times at 2-h intervals. After 7 days, the mice were sacrificed, and the brain tissues extracted and processed for immunoblotting, PT-PCR and immunohistochemistry.

### Injection of AAVs

The AAV9 viruses expressing mouse ARRB1 siRNA (AAV-siArrb1), mouse ARRB2 siRNA (AAV-siArrb2) or control siRNA (AAV-Ctrl) under the F4/80 promoter were microinjected bilaterally into C57BL/6J WT mice divided randomly into groups (male, 3–4 months old, *n* = 11 in each group) at the SNc (AP: −3.0 mm; ML: ± 1.3 mm; DV: −4.2 mm) at a rate of 0.2 μl/min in a stereotaxic apparatus. After 4 weeks, the mice were administered with MPTP to develop PD models.

### Isolation and treatment of primary cells

Primary microglia were isolated from the brain tissues of neonatal mice (within 3 days after birth) by treatment with 0.25% trypsin/EDTA as described [67]. The cells were plated into PLL-coated T75 flasks and cultured in DMEM/F-12 medium containing 1% penicillin/streptomycin and 10% FBS. The medium was changed every 3 days. After 10–14 days, the cells were split onto plates, incubated in serum-free base medium for 1 h, and treated with LPS (100 ng/ml) plus IFN-γ (20 ng/ml) at 37 °C for 24 h. The CM was collected and centrifuged at 12,000 *g* for 10 min at 4 °C and the supernatant was used to treat DA neurons.

BMDMs were isolated from the femur and tibia cavities of mice (male, 3 months old) and cultured in DMEM supplemented with 10% FBS, 1% streptomycin/penicillin, 1 mM sodium pyruvate, and 10 ng/ml GM-CSF as described [68]. The medium was changed every 3 days and the cells were used for further experiments after 7 days.

DA neurons were prepared from ventral mesencephalon of fetuses (E15–16) by treatment with 0.125% trypsin/EDTA as described previously [69]. The neurons were cultured in neurobasal medium supplemented with 2% B27 and 0.5 mM glutamine for 6 days and treated with microglial CM (microglia CM:neurobasal = 1:2) for 24 h.

### Plasmid construction

To generate ubiquitin-conjugated ARRB1 plasmids (ARRB1-Ub), a 228-bp DNA fragment encoding ubiquitin was subcloned into KpnI and AgeI sites of the ARRB1-EGFP vector. To generate ARRB2-Ub plasmids, a 1227-bp DNA fragment encoding ARRB2 was subcloned into HindIII and Kpn1 sites and ubiquitin into Kpn1 and BamH1 sites into the pEGFP-N1 vector. ARRB mutants, including ARRB1-S412A, ARRB1-S412D, ARRB2-S361A/T383A, and ARRB2-S361D/T383D, were generated by using QuikChange site-directed mutagenesis kits (Agilent, USA). All constructs were confirmed by DNA sequencing (GeneWiz, USA).

### Cell transfection

In knockdown experiments, primary cells were transfected with individual siRNAs targeting Nprl3 (Supplementary Table S1) using Lipofectamine RNAi MAX. In overexpression, phosphorylation and ubiquitination experiments, the cells were transfected with ARRB and Nprl3 plasmids using Lipofectamine 3000.

### Western blotting

Tissues or cells were lysed in RIPA buffer containing protease inhibitor cocktail. The lysates were centrifuged at 16,000 *g* for 15 min at 4 °C, and the supernatants were used for immunoblotting on 8–12% gels using antibodies against ARRB1 (1:800 dilution), ARRB2 (1:1000), iNOS (1:1000), CD206 (1:1000), p-p65 (1:1000), p65 (1:1000), p-IKKβ (1:1000), IKKβ (1:1000), p-STAT1 (1:1000), STAT1 (1:1000), p-ERK1/2 (1:1000), ERK1/2 (1:1000), Bcl-2 (1:500), Bax (1:1000), TH (1:2000), ARRB1 (1:500), ARRB2 (1:500), Nprl3 (1:1000), or β-actin (1:5000). The blots were analyzed using ImageQuant LAS 4000 imaging (GE Healthcare, USA) and Bio-Rad Gel Doc XR documentation systems.

### ELISA

The concentrations of cytokines, including TNF-α, IL-1β, and IL-6, in the supernatants of BMDMs were measured using ELISA kits, according to the manufacturer’s instructions.

### Quantitative RT-PCR

Total RNA extracted from mouse tissues or cells with TRIzol reagent was reverse transcribed with the TaKaRa Master Mix. The cDNAs obtained were mixed with FastStart Universal SYBR Green Master and gene-specific primers (Supplementary Table S2) for RT-PCR in a StepOnePlus instrument (Applied Biosystems, USA). GAPDH served as an internal control.

### Immunohistochemistry

Frozen 30-μm-thick midbrain sections or primary DA neurons were deparaffinized by treatment with 3% H_2_O_2_ for 30 min, blocked by 5% BSA plus 0.3% Triton X-100 for 60 min, and then incubated with anti-TH (1:1000) or anti-Iba-1(1:1000) antibodies at 4 °C overnight as described [66]. After washing, the slides were incubated with secondary antibodies (1:1000) for 60 min. After staining with the DAB system and imaging in a stereomicroscope (Olympus, Japan), the axon lengths were measured and the positive cells in the SNc were stereologically counted by Microbrightfield Stereo-Investigator software (Microbrightfield, USA).

### Immunofluorescence

Immunofluorescence staining was carried out as described previously [70]. Frozen 20-μm-thick brain sections and BMDMs were fixed with 4% PFA for 30 min, blocked with 5% BSA and then incubated with antibodies against CD16 (1:200), ARRB1 (1:50), ARRB2 (1:50), Iba-1 (1:1000), NeuN (1:800), or GFAP (1:800) at 4 °C overnight, followed by incubation with Alexa 594- or Alexa 555-conjugated secondary antibodies (1:500) for 1 h. The slides were stained with Gold antifade reagent with DAPI, and visualized under fluorescence microscopes (Nikon TE2000-S, Melville, NY or Zeiss LSM700, Oberkochen, Germany).

### Hoechst staining

Neurons were fixed with 4% paraformaldehyde (PFA) for 30 min and stained with Hoechst 33324 (1:1000 dilution) for 10 min. Apoptotic neurons were quantified by imaging in a fluorescence microscope (Olympus BX 60).

### Cell viability

The viability of primary neurons was measured using CCK-8, according to the manufacturer’s instructions.

### Co-IP

BMDMs were lysed in RIPA buffer containing protease inhibitors as described [68]. The cell extracts were pre-cleared with protein-A/G beads and then immunoprecipitated with 2 μg of antibodies against ARRB1 or ARRB2 at 4 °C overnight followed by incubation with protein A/G beads at 4 °C for 2.5 h. After washing for three times with RIPA buffer, the bound proteins were eluted and detected by immunoblotting.

### RNA-seq

Total RNA was extracted from primary microglia with TRIzol reagent. RNA-seq libraries were prepared and sequenced on an Illumina HiSeq 4000 system by Novogene. Differential expression analyses were performed by DESeq.

### Statistical analysis

Statistical tests were carried out using GraphPad Prism 7.0 software. Unpaired Student’s *t* test was used for comparison between two groups. One-way analysis of variance (ANOVA) or two-way repeated-measures ANOVA were used to assess differences among multiple groups. Data are presented as the means ± s.e. Differences were considered statistically significant at *P* < 0.05.

## Supplementary information

Supplementary Materials

Figure S1

Figure S2

Figure S3

Figure S4

Figure S5

Figure S6

Figure S7

Figure S8

Table S1

Table S2
