# Supplementary material for: Opposing functions of β-arrestin 1 and 2 in Parkinson’s disease via microglia inflammation and Nprl3
Source: Cell Death Differ. 2021 Mar 8;28(6):1822–36. doi: 10.1038/s41418-020-00704-9 (PMC8184754; doi:10.1038/s41418-020-00704-9)
Supplement: Supplementary file 1 — Supplementary Materials [file 41418_2020_704_MOESM1_ESM.docx]

**SUPPLEMENTARY MATERIALS**

**Fig. S1** Expression of ARRB1 and ARRB2 in astrocytes (**a**) and neurons (**b**) in the SNc of LPS-induced PD model mice. Similar results were obtained in 3 separate experiments. Scale bars, 50 µm.

**Fig. S2** Expression of ARRB1 and ARRB2 in the midbrain of WT, *Arrb1*^-/-^ and *Arrb2*^-/-^ mice. **a** Representative blots. **b** Quantitative data shown in (**a**). Quantitative data are mean ± s.e. (n = 3). NS, not significant.

**Fig. S3** Effects of ARRB1 or ARRB2 knockout on DA neuron death and microglia inflammation in PD models. **a-d** Expression of CD206 and iNOS in the midbrain of ARRB1 or ARRB2 knockout mice after LPS treatment (n = 3). **e-l** Immunohistochemistry (**e, g, i** and **k**) and stereological counts (**f**, **h**, **j** and **l**) of TH^+^ DA neuron (**e-h**) and Iba-1^+^ microglia (**i-l**) in the SNc of MPTP-induced PD models (n = 5). Scale bars, 200 µm (upper panels) or 40 µm (lower panels) in (**e**, **g**, **i** and **k**). **m** mRNA levels of pro- and anti-inflammatory markers in the midbrain of PD mice (n = 3). **n-q** Expression of CD206 and iNOS in the midbrain. **r-u** Expression of CD206 and iNOS in the midbrain of AAV-mediated microglial ARRB1 or ARRB2 knockdown mice after MPTP treatment (n = 3). Quantitative data are mean ± s.e.. **P* < 0.05, ***P* < 0.01, and ****P* < 0.001.

**Fig. S4** AAV-mediated depletion of ARRB1 and ARRB2 in microglia. **a** Schematic representation of the AAV9 virus construct used to express ARRB1, ARRB2 or control siRNA in microglia under the F4/80 promoter. **b** Expression of ARRB1 and ARRB2 in microglia in SNc of mice after AAV injection for 4 weeks. Scale bars, 50 µm. Similar results were obtained in 3 separate experiments.

**Fig. S5** Expression of ARRB1 and ARRB2 in microglia. **a-d** Expression of ARRB1 and ARRB2 in microglia after transfection with ARRB1 (**a-b**) or ARRB2 (**c-d**) for 24 h. Quantitative data are mean ± s.e. (n = 3). ***P* < 0.01.

**Fig. S6** Effect of phosphorylation and ubiquitination of ARRB1 and ARRB2 on microglia inflammation. Microglia were transfected with ARRB1, ARRB1-S412A, ARRB1-S412D, ARRB1-Ub, ARRB2, ARRB2-S361A/T383A, ARRB2-S361D/T383D or ARRB2-Ub and the levels of pro-inflammatory gene transcripts were measured after LPS plus IFN-γ stimulation for 6 h. Quantitative data are mean ± s.e. (n = 4). ****P* < 0.001 vs respective control; ^#^*P* < 0.05, ^##^*P* < 0.01 and ^###^*P* < 0.001 vs cells transfected with vector and stimulated with LPS plus IFN-γ.

**Fig. S7** Analysis of RNA-seq. **a** Heatmap of normalized read counts from WT or Arrb2^-/-^ microglia treated with LPS plus IFN-γ for 6 h. The total RNA was sequenced on an Illumina HisSeq 4000. **b** A volcano plot of gene changes in microglia from *Arrb2^-/-^* mice as compared with those from WT mice after LPS plus IFN-γ treatment. Among 130 upregulated and 56 downregulated genes, the top 10 enriched BPs were determined. The intensity of the red color indicates the significance of BPs. **c** Enriched KEGG pathways found among the upregulated genes in microglia from *Arrb2^-/-^* mice as compared with WT mice.

**Fig. S8** Nprl3 expression in microglia after transfection with NPRL3 for 24 h (**a** and **b**) or Nprl3 siRNA for 48 h (**c** and **d**). Quantitative data are mean ± s.e. (n = 3). ****P* < 0.001.
