# Supplementary figures and images for: Opposing functions of β-arrestin 1 and 2 in Parkinson’s disease via microglia inflammation and Nprl3
Source: Cell Death Differ. 2021 Mar 8;28(6):1822–36. doi: 10.1038/s41418-020-00704-9 (PMC8184754; doi:10.1038/s41418-020-00704-9)

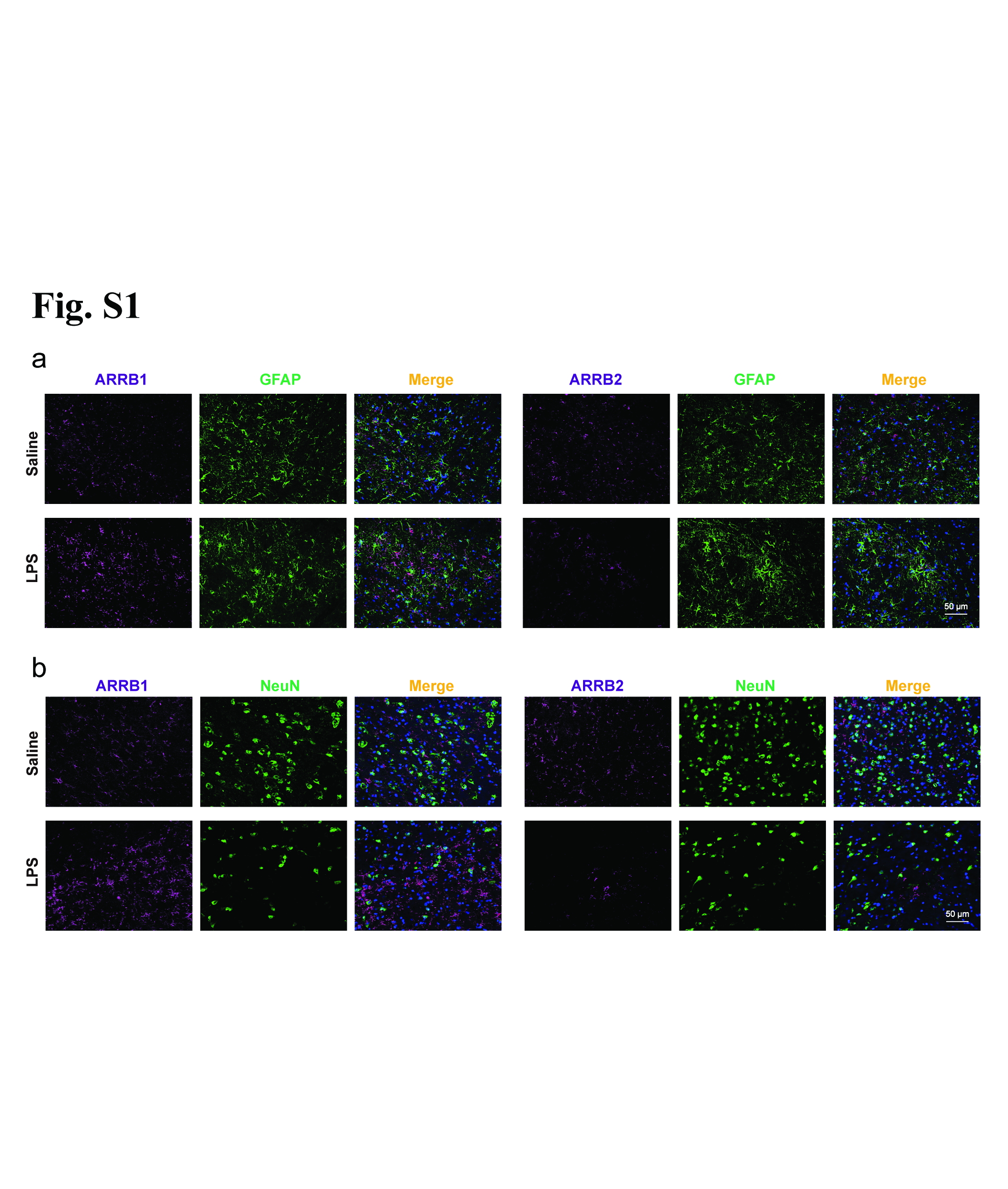

Supplement: Supplementary file 2 — Figure S1 [file 41418_2020_704_MOESM2_ESM.tif]

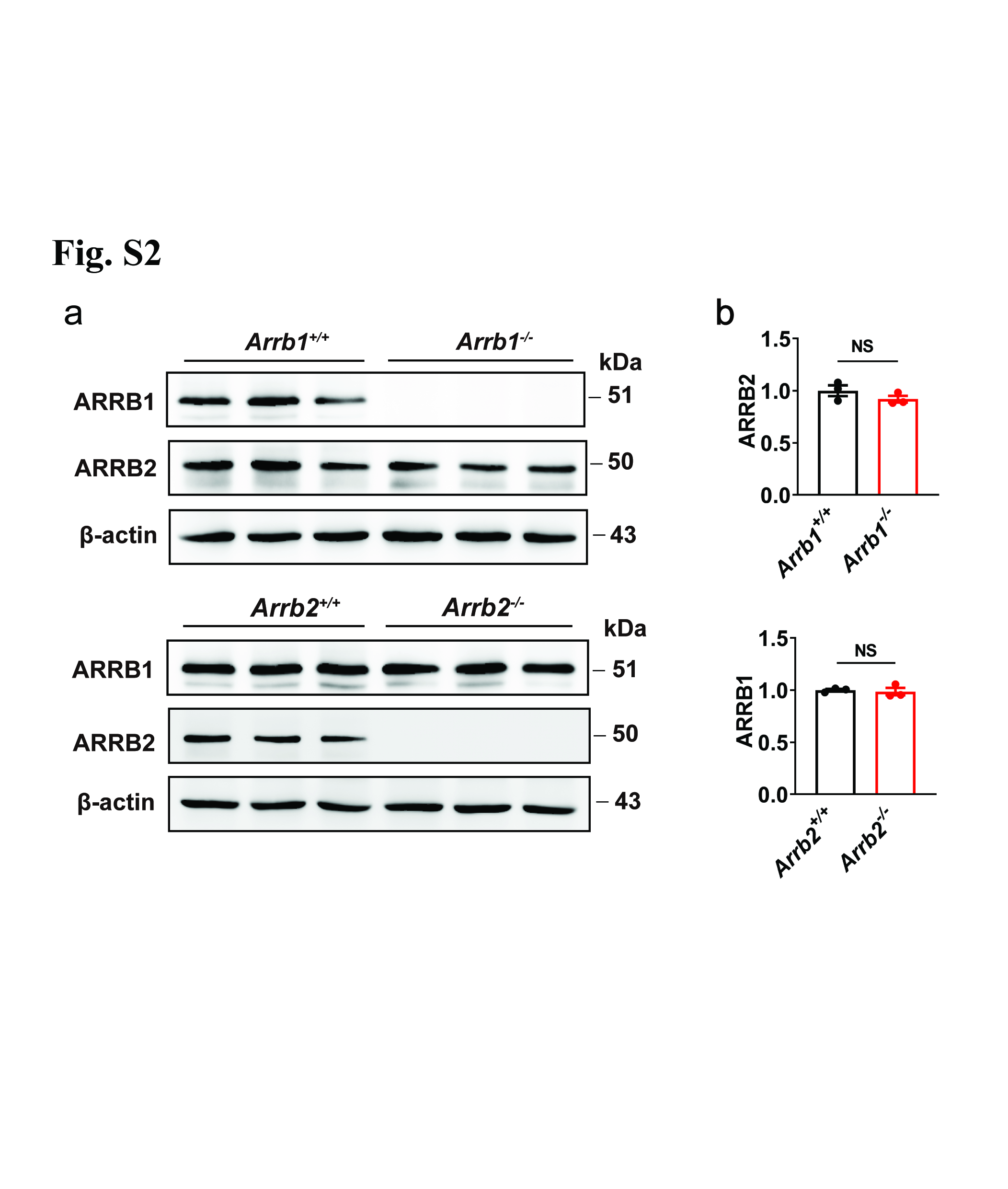

Supplement: Supplementary file 3 — Figure S2 [file 41418_2020_704_MOESM3_ESM.tif]

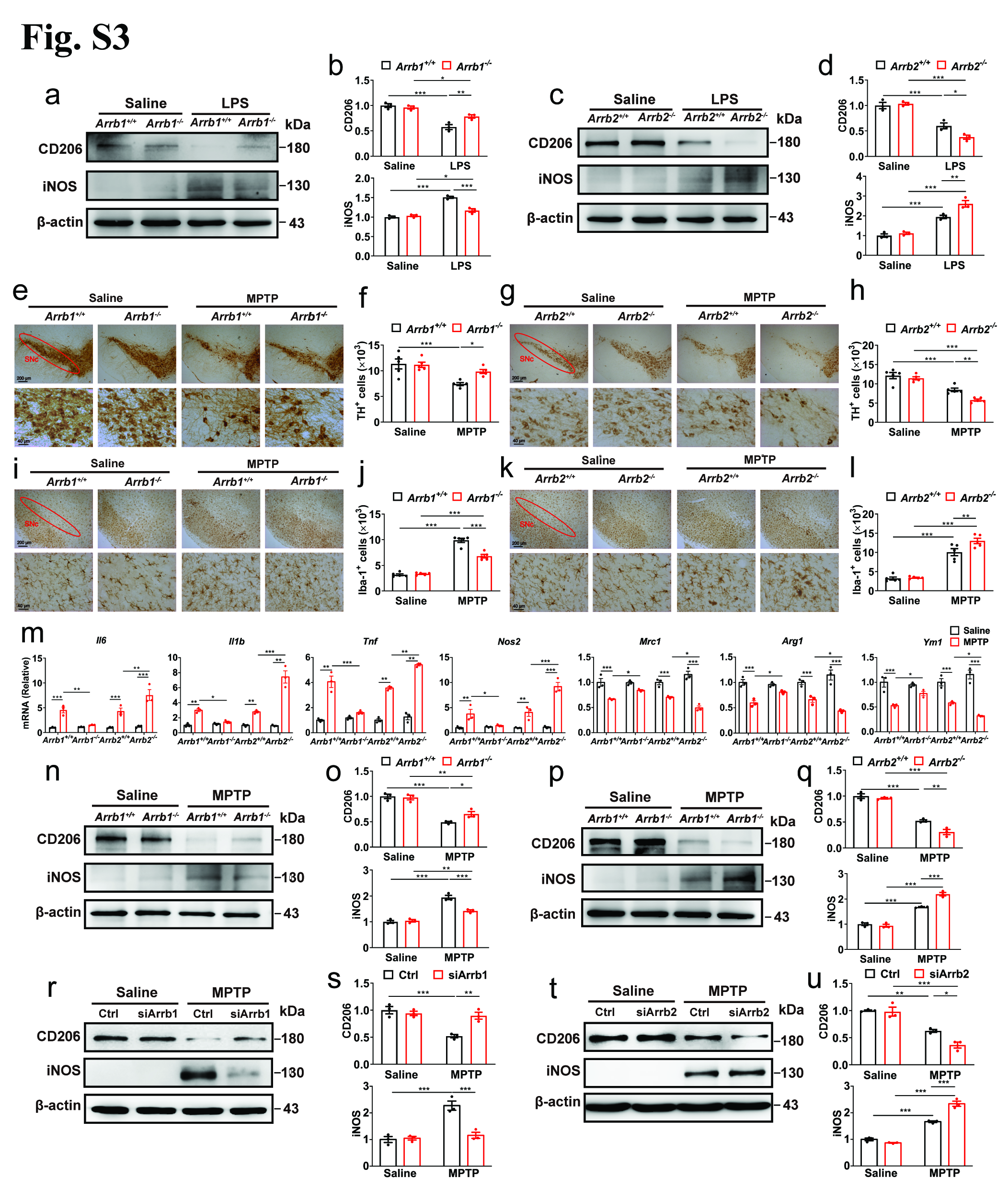

Supplement: Supplementary file 4 — Figure S3 [file 41418_2020_704_MOESM4_ESM.tif]

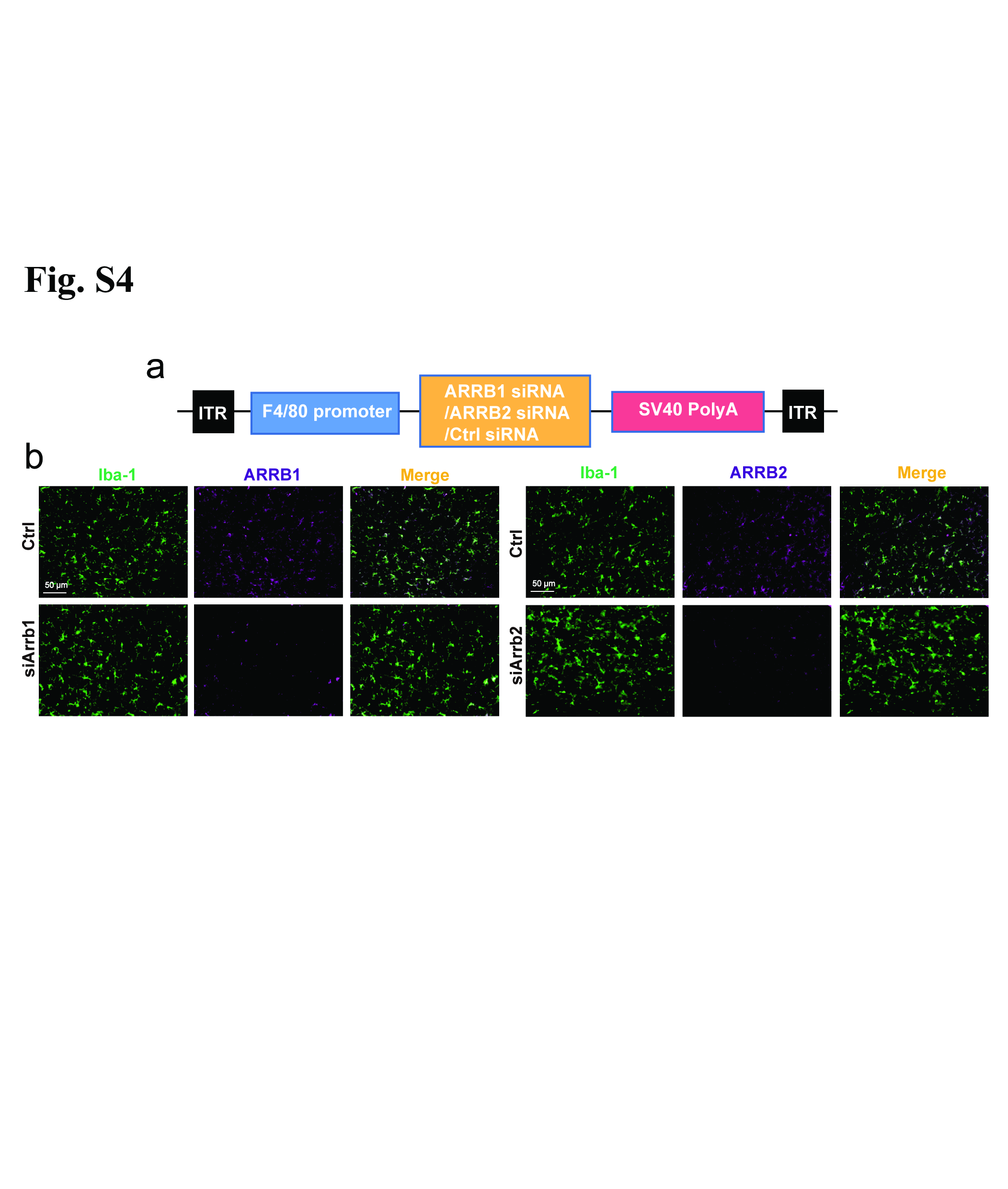

Supplement: Supplementary file 5 — Figure S4 [file 41418_2020_704_MOESM5_ESM.tif]

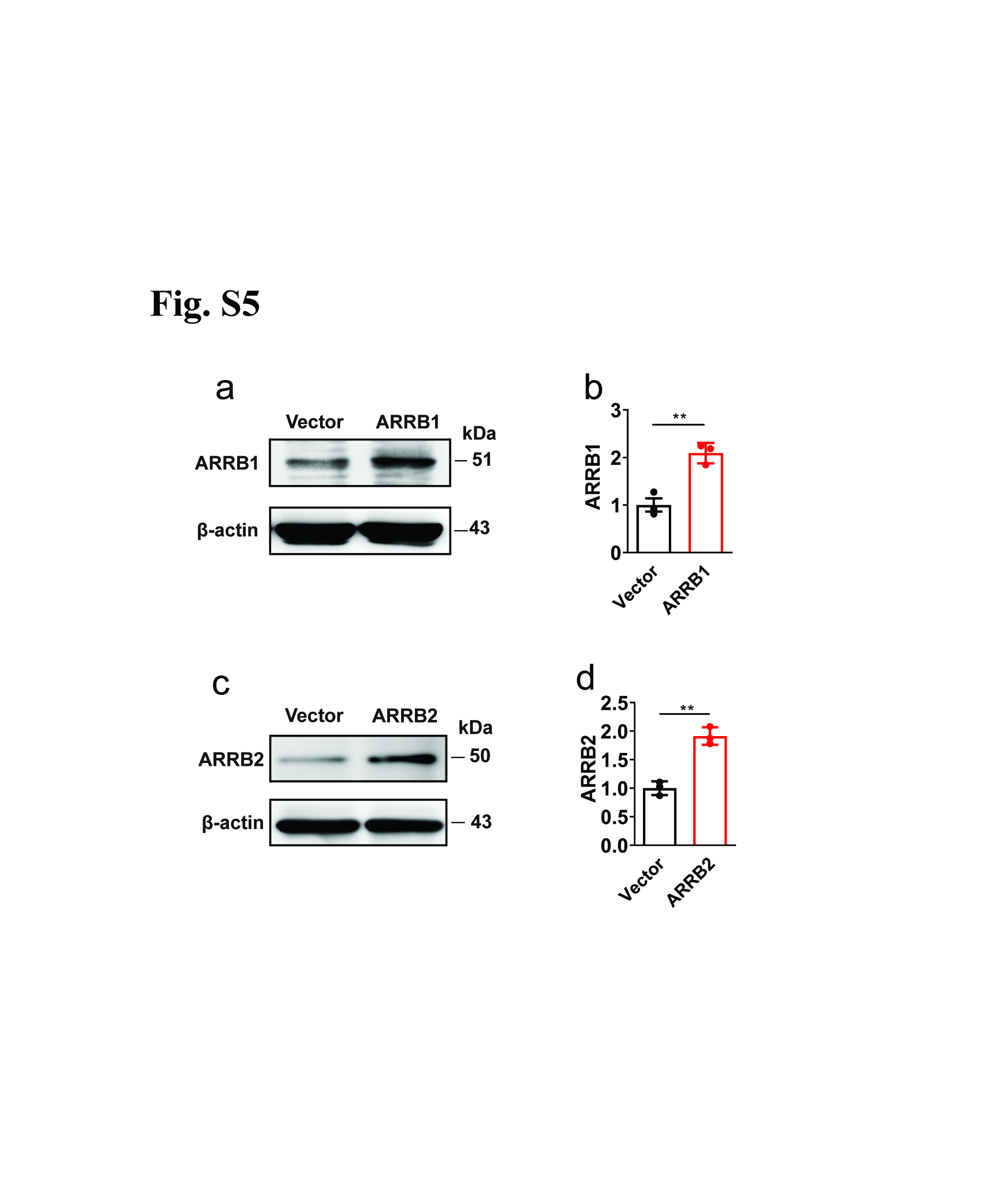

Supplement: Supplementary file 6 — Figure S5 [file 41418_2020_704_MOESM6_ESM.tif]

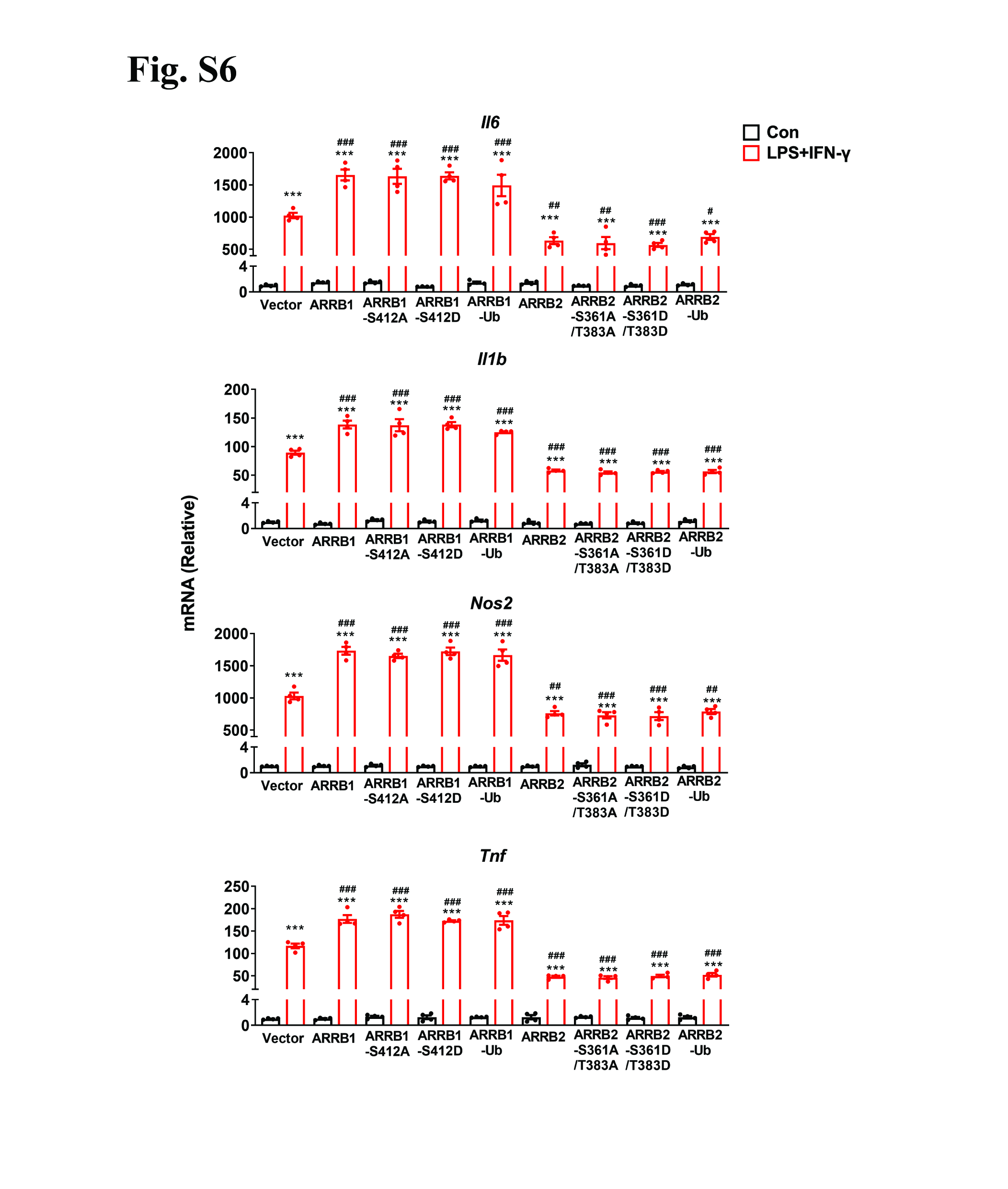

Supplement: Supplementary file 7 — Figure S6 [file 41418_2020_704_MOESM7_ESM.tif]

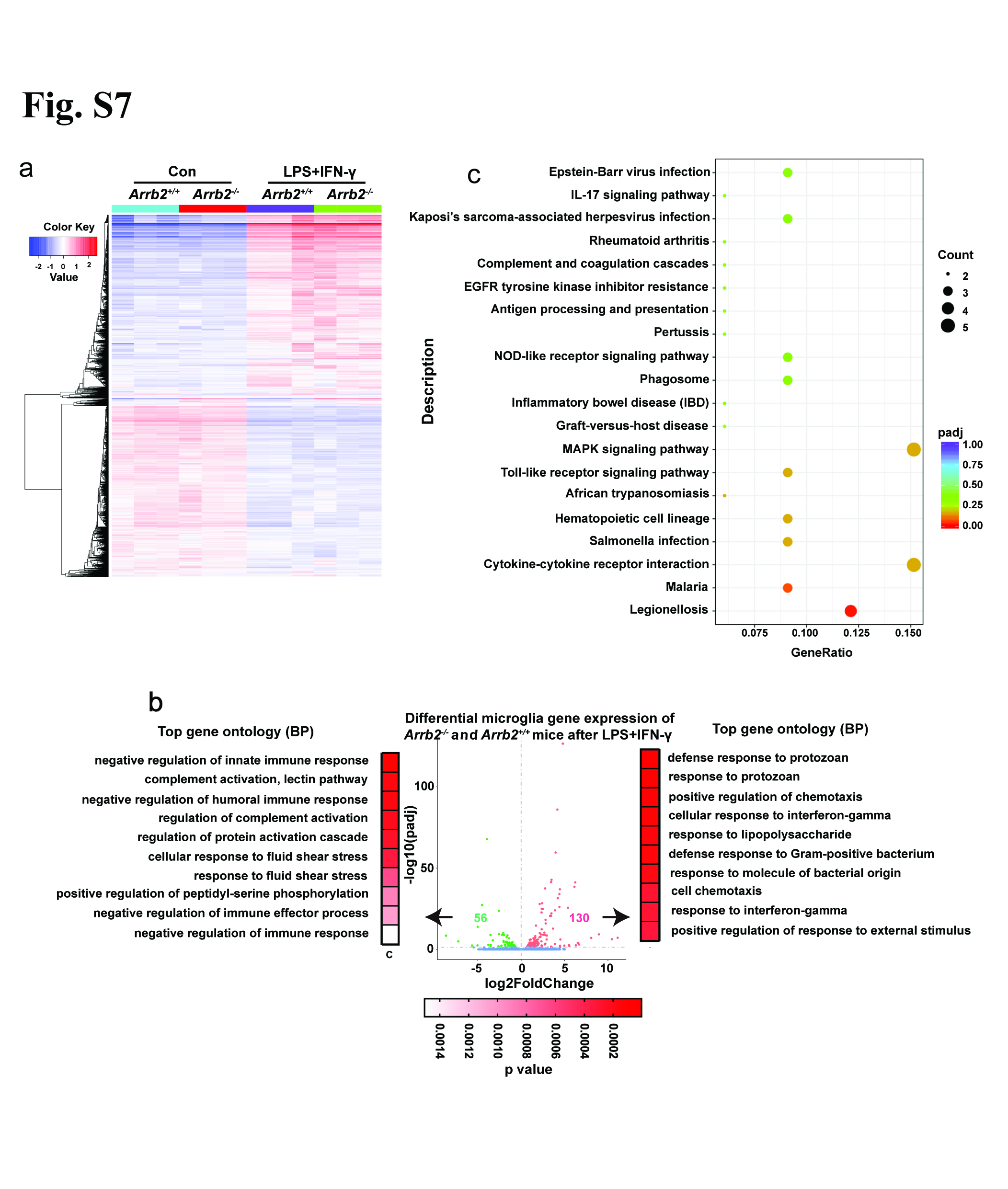

Supplement: Supplementary file 8 — Figure S7 [file 41418_2020_704_MOESM8_ESM.tif]

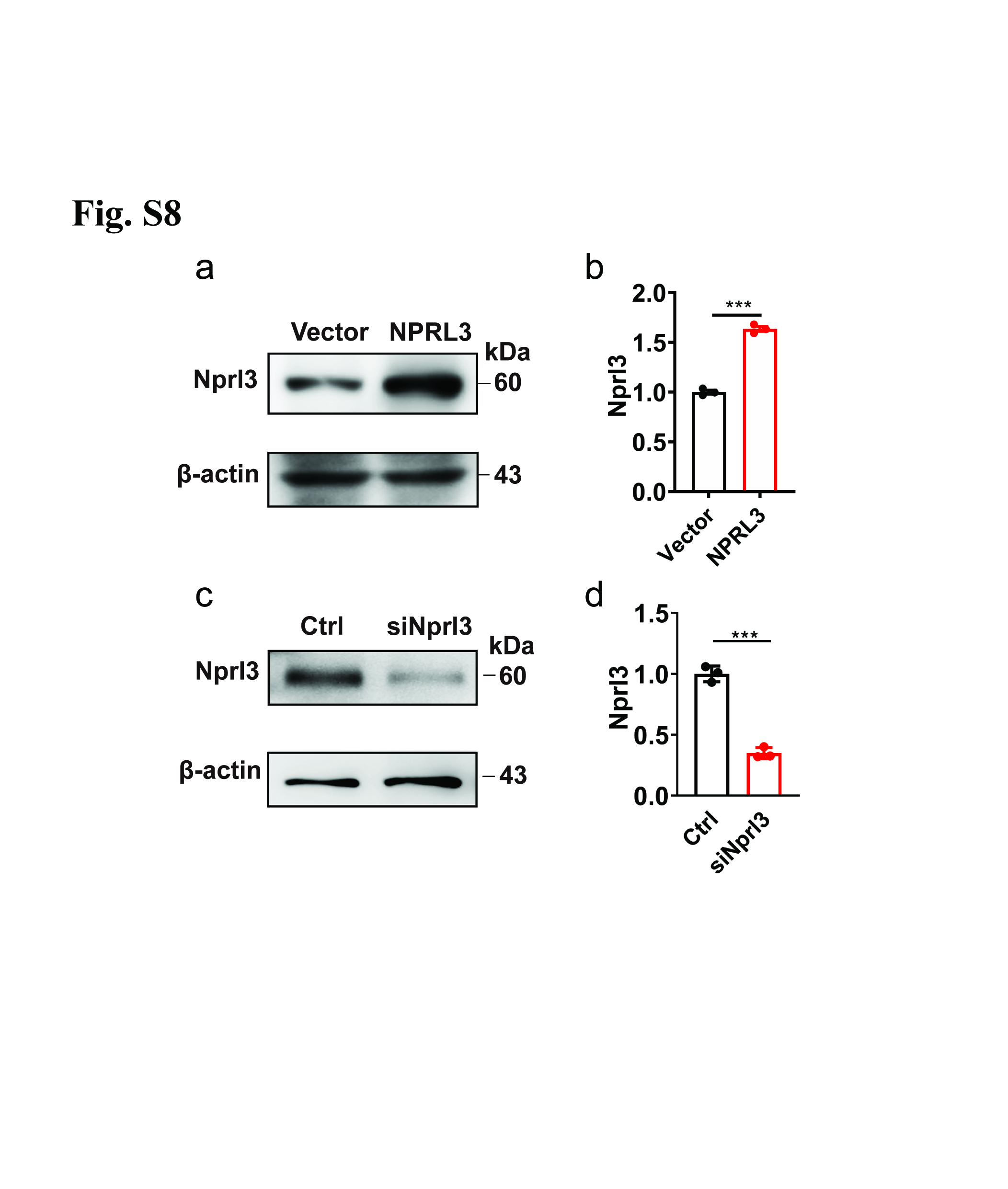

Supplement: Supplementary file 9 — Figure S8 [file 41418_2020_704_MOESM9_ESM.tif]
