# Supplementary material for: Opposing functions of β-arrestin 1 and 2 in Parkinson’s disease via microglia inflammation and Nprl3
Source: Cell Death Differ. 2021 Mar 8;28(6):1822–36. doi: 10.1038/s41418-020-00704-9 (PMC8184754; doi:10.1038/s41418-020-00704-9)
Supplement: Supplementary file 10 — Table S1 [file 41418_2020_704_MOESM10_ESM.docx]

| **Table S1: siRNA duplexes used to knockdown specific proteins** | | | |  |
| --- | --- | --- | --- | --- |
| Control | sense | UUCUCCGAACGUGUCACGUTT |  | |
|  | antisense | ACGUGACACGUUCGGAGAATT |  | |
| ARRB1 | sense | CCUGGUGGAUCCUGAGUAUTT |  | |
|  | antisense | AUACUCAGGAUCCACCAGGTT |  | |
| ARRB2 | sense | GCUUGUGGAGUAGACUUUGTT |  | |
|  | antisense | CAAAGUCUACUCCACAAGCTT |  | |
| Nprl3 | sense | GCAAGGCUGUCAUCAUCUATT |  | |
|  | antisense | UAGAUGAUGACAGCCUUGCTT |  | |
|  |  |  | |  |
|  |  |  | |  |
