# Supplementary material for: Opposing functions of β-arrestin 1 and 2 in Parkinson’s disease via microglia inflammation and Nprl3
Source: Cell Death Differ. 2021 Mar 8;28(6):1822–36. doi: 10.1038/s41418-020-00704-9 (PMC8184754; doi:10.1038/s41418-020-00704-9)
Supplement: Supplementary file 11 — Table S2 [file 41418_2020_704_MOESM11_ESM.docx]

| **Table S2: Primers used for RT-PCR and plasmid construction** | | |
| --- | --- | --- |
| TNF-α | 5'-TTGCTCTGTGAAGGGAATGG-3'  5'-GGCTCTGAGGAGTAGACAATAAAG-3' |  |
| IL-1β | 5'-TCAGGCAGGCAGTATCACTC-3'  5'-CATGAGTCACAGAGGATGGG-3' |  |
| Il12rb1 | 5’-CGAATTGGACCTTGGGTGAC-3’  5’-TGGATCTCTTGGGCCATGTT-3’ |  |
| Lpar1 | 5’-CAACCTGGTGACCTTTGTGG-3’  5’-GGTCCAGAACTATGCCGAGA-3’ |  |
| Tom1l1 | 5’-TGTGGCCCGAGTTTCCAATC-3’  5’-AGCGGTAAGGTGTATCTGGGA-3’ |  |
| Cd14 | 5’-CTGCCCTCTCCACCTTAGAC-3’  5’-TAGTCCTTGCAGCTGTACCC-3’ |  |
| Tmem100 | 5’-GACAATGGAGAAAAACCCCAAGA-3’  5’-GGTAGCAGGAGAGTTCGGC-3’ |  |
| Gpat3 | 5’-CCTCCTCACACGAACCAATGT-3’  5’-CAATAGCGCACTAGGACACCC-3’ |  |
| Rps2 | 5’-CCAGGTTCAAGGCTTTCGTC-3’  5’-TGGGGCTTGCCAATCTTGTT-3’ |  |
| Otx1 | 5’-ACCCACTGAGCCAATCTTCA-3’  5’-CAGACAGTCGGGAGAGTTGA-3’ |  |
| IL-6 | 5'-ATCCAGTTGCCTTCTTGGGACTGA-3'  5'-TAAGCCTCCGACTTGTGAAGTGGT-3' |  |
| iNOS | 5'-GAACGGAGAACGTTGGATTTG-3'  5'-TCAGGTCACTTTGGTAGGATTT-3' |  |
| S100a10 | 5’-CCTCTGGCTGTGGACAAAAT-3’  5’-CTGCTCACAAGAAGCAGTGG-3’ |  |
| Nes | 5’-AGAGTCAGATCGCTCAGATCC-3’  5’-GCAGAGTCCTGTATGTAGCCAC-3’ |  |
| Nprl3 | 5’-ATTGAACGGAGCCTGAAAGC-3’  5’-CAGGTTCTTCACAGCAGACG-3’ |  |
| Cd5l | 5’-TGGGCAAGATGTCGTGTTCTG-3’  5’-CCAGGAACCCTTGTGTAGCA-3’ |  |
| Pttg1 | 5’-CGAGTCGGCAAAGTGTTCAA-3’  5’-GGCAACTCTGTTGACTGTCC-3’ |  |
| Tfpi | 5’-GGGATGTGAAGGGAACGAGA-3’  5’-CAGAGTCCAGGGTCCTCTTC-3’ |  |
| Otx1 | 5’-ACCCACTGAGCCAATCTTCA-3’  5’-CAGACAGTCGGGAGAGTTGA-3’ |  |
| GAPDH | 5'-AACGACCCCTTCATTGAC-3'  5'-TCCACGACATACTCAGCAC-3' |  |
| ARRB1-Ub | 5'-GATCGGTACCGATGCAGATCTTCGTGAAG-3'  5'-GATCACCGGTCTACCCACCTCTGAGACGGAG-3' |  |
| ARRB2 | 5'-GATCAAGCTTATGGGGGAGAAACCCGGG-3'  5'-GATCGGTACCGCAGAACTGGTCGTCATAGTC-3' |  |
| ARRB2-Ub | 5'-GATCGGTACCGATGCAGATCTTCGTGAAG-3'  5'-GATCGGATCCCTACCCACCTCTGAGACGGAG-3' |  |
| ARRB1-S412A | 5'-ACGGCACCGGCGCTCCGCACCTC-3'  5'-GAGGTGCGGAGCGCCGGTGCCGT-3' |  |
| ARRB1-S412D | 5'-GATGACGGCACCGGCGATCCGCACCTCAACAA-3'  5'-TTGTTGAGGTGCGGATCGCCGGTGCCGTCATC-3' |  |
| ARRB2-S361A | 5'-CCCAGGCCCCAGGCAGCTGTTCCTGA-3'  5'-TCAGGAACAGCTGCCTGGGGCCTGGG-3' |  |
| ARRB2-S361D | 5'-CCCTTCCCAGGCCCCAGGATGCTGTTCCTGAAACAGA-3'  5'-TCTGTTTCAGGAACAGCATCCTGGGGCCTGGGAAGGG-3' |  |
| ARRB2-T383A | 5'-CGAAACCAACTATGCCGCAGACGACGACATCGT-3'  5'-ACGATGTCGTCGTCTGCGGCATAGTTGGTTTCG-3' |  |
| ARRB2-T383D | 5'-GAATTCGAAACCAACTATGCCGATGACGACGACATCGTGTTTGAG-3'  5'-CTCAAACACGATGTCGTCGTCATCGGCATAGTTGGTTTCGAATTC-3' |  |
